# Correction: *Toxoplasma gondii* Infection in Alpine Red Deer (*Cervus elaphus*): Its Spread and Effects on Fertility

**DOI:** 10.1371/journal.pone.0142357

**Published:** 2015-10-30

**Authors:** Nicoletta Formenti, Tiziana Trogu, Luca Pedrotti, Alessandra Gaffuri, Paolo Lanfranchi, Nicola Ferrari

## Notice of Republication

This article was republished on October 21, 2015, to correct the spelling of “*Cervus elaphus*” in the title, abstract, and citation. Please download this article again to view the correct version. The originally published, uncorrected article and the republished, corrected article are provided here for reference.

## Supporting Information

S1 File Originally published, uncorrected article. (PDF)

S2 File Republished, corrected article. (PDF)
