# Supplementary material for: Toxoplasma gondii Infection in Alpine Red Deer (Cervus elaphus): Its Spread and Effects on Fertility
Source: PLoS One. 2015 Sep 25;10(9):e0138472. doi: 10.1371/journal.pone.0138472 (PMC4583299; doi:10.1371/journal.pone.0138472)
Supplement: S1 Table — (DOCX) [file pone.0138472.s001.docx]

|  | **females** | **males** |
| --- | --- | --- |
| **calves (<1 year-old)** | 7 | 14 |
| **yearlings (1 year-old)** | 9 | 12 |
| **adults (> 2 year-old)** | 34 | 5 |
| **Tot** | 50 | 31 |
