# Supplementary material for: Toxoplasma gondii Infection in Alpine Red Deer (Cervus elaphus): Its Spread and Effects on Fertility
Source: PLoS One. 2015 Sep 25;10(9):e0138472. doi: 10.1371/journal.pone.0138472 (PMC4583299; doi:10.1371/journal.pone.0138472)
Supplement: S2 Table — (DOCX) [file pone.0138472.s002.docx]

|  |  | **Coeff.** | **Wald Chi-Square** | **df** | **P value** |
| --- | --- | --- | --- | --- | --- |
| **(Intercept)** |  | 0.198 | 8.794 | 1 | 0.003 |
| **Area** |  |  | 3.547 | 1 | 0.060 |
|  | area 1 | -1.623 |  |  |  |
|  | area 2 | 0 |  |  |  |
| **Age class** |  |  | 8.980 | 2 | 0.011 |
|  | calves | -3.067 |  |  |  |
|  | yearlings | 0.262 |  |  |  |
|  | adults | 0 |  |  |  |
