# Supplementary material for: Toxoplasma gondii Infection in Alpine Red Deer (Cervus elaphus): Its Spread and Effects on Fertility
Source: PLoS One. 2015 Sep 25;10(9):e0138472. doi: 10.1371/journal.pone.0138472 (PMC4583299; doi:10.1371/journal.pone.0138472)
Supplement: S3 Table — (DOCX) [file pone.0138472.s003.docx]

|  |  | **Coeff.** | **Wald Chi-Square** | **df** | **P value** |
| --- | --- | --- | --- | --- | --- |
| **(Intercept)** |  | 0.585 | 62.197 | 1 | <0.001 |
| **Age class** |  |  | 4.753 | 1 | 0.029 |
|  | yearlings | 0.447 |  |  |  |
|  | adults | 0 |  |  |  |
