# Supplementary material for: Toxoplasma gondii Infection in Alpine Red Deer (Cervus elaphus): Its Spread and Effects on Fertility
Source: PLoS One. 2015 Sep 25;10(9):e0138472. doi: 10.1371/journal.pone.0138472 (PMC4583299; doi:10.1371/journal.pone.0138472)
Supplement: S4 Table — (DOCX) [file pone.0138472.s004.docx]

|  |  | **Coeff.** | **Wald Chi-Square** | **df** | **P value** |
| --- | --- | --- | --- | --- | --- |
| **(Intercept)** |  | 32.017 | 103.833 | 1 | <0.001 |
| **Area** |  |  | 19.149 | 1 | <0.001 |
|  | area 1 | -15.215 |  |  |  |
|  | area 2 | 0 |  |  |  |
| **Age class** |  |  | 2.209 | 2 | 0.331 |
|  | 2-3 year-old | -3.641 |  |  |  |
|  | 4-6 year-old | 0.827 |  |  |  |
|  | >7 year-old | 0 |  |  |  |
| **Serological titres** |  | 1.209 | 25.641 | 1 | <0.001 |
| **Sampling day** |  | 1.257 | 45.004 | 1 | <0.001 |
| **KFI** |  | 0.076 | 23.930 | 1 | <0.001 |
| **Serological titres:Area** |  |  | 25.412 | 1 | <0.001 |
|  | serological titres:area 1 | -29.957 |  |  |  |
|  | serological titres:area 2 | 0 |  |  |  |
| **Serological titres:Age class** |  |  | 11.049 | 2 | 0.004 |
|  | serological titres:2-3 year-old | -14.051 |  |  |  |
|  | serological titres:4-6 year-old | 2.092 |  |  |  |
|  | serological titres:>7 year-old | 0 |  |  |  |
