# Supplementary material for: Toxoplasma gondii Infection in Alpine Red Deer (Cervus elaphus): Its Spread and Effects on Fertility
Source: PLoS One. 2015 Sep 25;10(9):e0138472. doi: 10.1371/journal.pone.0138472 (PMC4583299; doi:10.1371/journal.pone.0138472)
Supplement: S1 Text — (PDF) [file pone.0138472.s005.pdf]

PARCO
NAZIONALE
DELLO
STELVIO
NATIONAL
PARK
STILFSER
JOCH

Dr Nicoletta Formenti
Department of Veterinary Sciences and Public Health
Università degli Studi di Milano
Via Celoria 10
20133 Milano - Italy

Prot. n. 1962 del 16/07/2015

REFERENCE: PLOS ONE - Submission PONE-D-15-18187R1 - [EMID:d68af399800e7704].

Dear Dr Formenti,

The Stelvio National Park, the original copyright holder of all the data in Figure 1 of the submitted paper PONE-D-15-18187R1 - [EMID:d68af399800e7704], allows the permission for the open-access journal PLOS ONE to publish Figure 1 under the Creative Commons Attribution License (CCAL) CC BY 3.0.

Best regards

CONSORZIO PARCO NAZIONALE DELLO STELVIO KONSORTIUM NATIONALPARK STILFSER JOCH

THE SCIENTIFIC COORDINATOR
Dr Luca Pedrotti

Consorzio del Parco Nazionale dello Stelvio
Via De Simoni, 42 - 23032 Bormio SO
Tel. 0342 900811 - Fax 0342 900898
Partita IVA 00685060147
Internet: www.stelviopark.it
E-mail: info@stelviopark.it
