# Supplementary material for: Correction: Toxoplasma gondii Infection in Alpine Red Deer (Cervus elaphus): Its Spread and Effects on Fertility
Source: PLoS One. 2015 Oct 30;10(10):e0142357. doi: 10.1371/journal.pone.0142357 (PMC4627810; doi:10.1371/journal.pone.0142357)
Supplement: S2 File — (PDF) [file pone.0142357.s002.pdf]

RESEARCH ARTICLE

Toxoplasma gondii Infection in Alpine Red Deer (*Cervus elaphus*): Its Spread and Effects on Fertility

Nicoletta Formenti[1]*, Tiziana Trogu[1], Luca Pedrotti[2], Alessandra Gaffuri[3], Paolo Lanfranchi[1], Nicola Ferrari[1]

1 Department of Veterinary Sciences and Public Health, Università degli Studi di Milano, Milan, Italy, **2** Consorzio Parco Nazionale dello Stelvio, Bormio (Sondrio), Italy, **3** Istituto Zooprofilattico Sperimentale della Lombardia e dell'Emilia Romagna ''Bruno Ubertini'', Bergamo, Italy

* nicoletta_formenti@yahoo.it

OPEN ACCESS

Citation: Formenti N, Trogu T, Pedrotti L, Gaffuri A, Lanfranchi P, Ferrari N (2015) *Toxoplasma gondii* Infection in Alpine Red Deer (*Cervus elaphus*): Its Spread and Effects on Fertility. PLoS ONE 10(9): e0138472. doi:10.1371/journal.pone.0138472

Editor: Guido Favia, University of Camerino, ITALY

Received: April 28, 2015

Accepted: August 31, 2015

Published: September 25, 2015

Copyright: © 2015 Formenti et al. This is an open access article distributed under the terms of the Creative Commons Attribution License, which permits unrestricted use, distribution, and reproduction in any medium, provided the original author and source are credited.

Data Availability Statement: All relevant data are within the paper and its Supporting Information files.

Funding: This research was supported by a grant from Progetto Giovani 2009 "Valorizzazione dei prodotti tipici dell'agroalimentare e sicurezza alimentare attraverso nuovi sistemi di caratterizzazione e garanzia di qualità" (PL) and partially by the Italian Ministry of University and Research (PRIN project n. 2010P7LFW4) (PL). The funders had no role in study design, data collection and analysis, decision to publish, or preparation of the manuscript.

Abstract

In contrast to the depth of knowledge on the pathological effects of parasitism in domestic animals, the impact of the vast majority of parasites on wildlife hosts is poorly understood and, besides, information from domestics is rarely usable to disclose the parasites' impact on free-ranging populations' dynamics. Here we use *Toxoplasmosis* as a study model since, until now, the infection process and the protozoan's effects in natural conditions has received little attention. We analysed 81 sera from red deer (*Cervus elaphus*) sampled in Italian Alps and through generalized linear models we evaluated (1) the epidemiological factors influencing *T. gondii* infection dynamics; (2) its impact on female fertility. High seroprevalence of *T. gondii* infection was recorded in yearling (1 year-old; prevalence = 52.4%) and adult (>2 year-old; prevalence = 51.3%) red deer, while calves (<1 year-old) did not contract the infection suggesting horizontal transmission as the main route of infection. The stable prevalence between yearlings and adults and the higher serological titres of younger individuals lead to two alternative infection processes suggesting a difference between age classes or in acquiring the infection or in responding to the pathogen. No associations between *T. gondii* serological titres and pregnancy status was observed indicating no direct effect on the probability of being pregnant; nevertheless a relation between females' higher serological titres and lower foetal development emerged, suggesting potential effects of the parasite infection on deer reproduction. The results demonstrate high seroprevalence of *T. gondii* infection in free-ranging red deer and, furthermore, the effect on foetal development suggests the potential impact of the parasite on red deer fertility and thus on its population dynamics.

Introduction

Parasite infections may affect the dynamics of free-ranging animal populations [1–3] through effects on survival [4] or fecundity [5]. However, although the impact of some parasite species

Competing Interests: The authors have declared that no competing interests exist.

have been determined in wildlife [6], the effect of the vast majority is still undefined. Medical veterinary sciences has developed a broad knowledge base regarding the pathological effects of parasitism on domestic animal hosts [7–9]. Unfortunately this information is rarely transposed to wildlife and scarcely usable to disclose the parasite impact on free-ranging populations' dynamics. Here we use *Toxoplasmosis* as a study model.

Toxoplasma gondii is known to infect a wide spectrum of animals as intermediate hosts [10–18] and its pathological effects are well known in many of these species [19–22]. In humans *T. gondii* represents a threat for pregnant women and immunocompromised patients [23–25] while in livestock, besides their potential role in the protozoan's transmission to humans, *T. gondii* can affect reproductive performance, with obvious economic consequences [26–27]. Nevertheless the infection dynamics and effects of *T. gondii* in free-ranging ungulates has received little attention. Several ungulate species are reported as intermediate hosts [28–33] but little is known about its effects on population dynamics and regarding epidemiological factors (host and environmental) influencing *T. gondii* infection process[34] in these species. Moreover we are not aware of studies on *T. gondii*–associated abortion or reproductive disorders in red deer (*Cervuselaphus*) and, although this species appears to acquire *T. gondii* infection only temporarily and eliminate it in early adulthood [24], the vertical transmission recorded in white tailed deer (*Odocoileus virginianus*) [35] and the natural transplacental toxoplasmosis shown in a stillborn reindeer (*Rangifer tarandus*) foetus [36] suggest that red deer could also be affected.

Here we carried out a sero-epidemiological investigation in free-ranging red deer from Italian Alps aimed to investigate: (1) host and environmental factors influencing the infection dynamics within the population; (2) if the pathogen may impact on female fertility causing (i) early abortion or (ii)effects on the physiological foetal development.

Materials and Methods

Ethics Statement

This research did not involve purposeful killing of animals. All samples were gathered from dead free-ranging red deer legally shot by hunters during the depopulation management plan launched by the Stelvio National Park. Thus, no animals were killed specifically for this study. The plan was approved by the Italian Ministry of the Environment and Protection of Land and Sea and by The Institute for Environmental Protection and Research (ISPRA) and it was authorized by Lombardia Region (decree n° 387, 2/12/2011). Therefore the sampling and the study of the gathered material (blood serum) did not require an additional approval of the ethics committee.

Study area

The study area lies in Central Italian Alps (Stelvio National Park; Long 10,41; Lat 46,48)at an altitude between 1300 and 2400 m a.s.l.. The very high population density of red deer recorded in this area led the National Park to launch a management plan to reduce deer density and their impact on forest regeneration and biodiversity. The study area was divided into two different macro-areas, 1 and 2 (respectively 772 ha and 707 ha wide) (Fig 1), in which radio-tracking has shown that deer movements between them does not occur although not physical barrier to transit exists. In area 1 "low anthropised" human settlements and agricultural landscapes (i.e. hay meadows around small villages) constitute a small fraction of the whole surface (7%)in contrast with area 2 "high anthropised" in which they are much more widely distributed and represent 32% of the total surface.

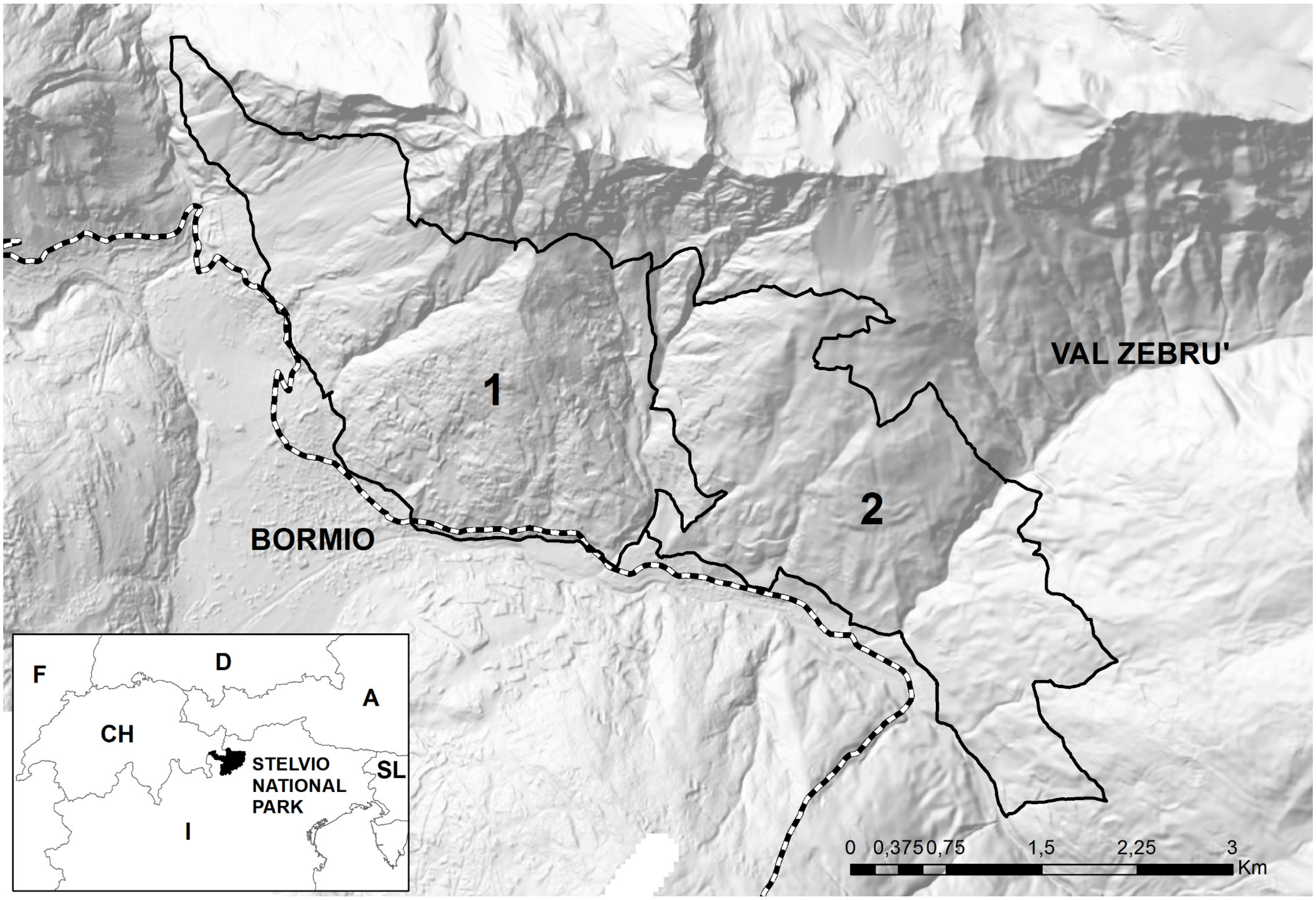

Fig 1. Representation of the two sampling areas (black continuous line), 1 and 2; dotted line: limits of Stelvio National Park. Reprinted from the Stelvio National Park Information System under a CC BY license, with permission from Stelvio National Park, original copyright 2015 (S1 Text).

doi:10.1371/journal.pone.0138472.g001

Sampling

During two consecutive weeks between the end of November and the beginning of December 2012, 81 red deer sera (S1 Table) were collected from subjects culled during the depopulation management plan. For each subject age, sex, shooting area and body condition (i.e. Kidney Fat Index (KFI)) were registered. Since age is known to influence female fertility [37–38], adult females (> 2 years-old) were further grouped into three age classes (2–3 year-old, 4–6 year-old and >7 year-old). Moreover lactation (lactation present = 24; lactation absent = 10) and pregnancy (pregnant = 27; non pregnant = 7) were also registered. For each pregnant female, foetal morphometric measures (body weight and length)were recorded to determine the foetal development(gestational ages) according to[39].

Serological investigation

Sera were tested for the presence of anti-*T. gondii* IgG using a commercial ELISA kit (ID Screen Toxoplasmosis Indirect ELISA, IDVET, Montpellier, France),validated for ruminants, with known sensitivity and specificity of respectively 91.3% and 98.7% [40]. The validation study of

the ELISA test was previously conducted by the manufacturer (IDVET, Montpellier, France) providing values of the coefficient of variation (CV%). In particular, repeatability (intra-assay repeatability) was evaluated by measuring the CV% of 96 repetitions of the positive control and a weak positive serum. The measured CV% was 6% and 8%, respectively. Reproducibility (inter-assay repeatability) was evaluated by performing the intra-plate repeatability assay on two separate runs. The CV% obtained was 7% for the positive control and 9% for the weak positive serum. Therefore, we conducted the test according to the manufacturer's instructions, as previously performed by [33] for red deer and by Other Authors for domestic and wild animals [41–46]. The presence and quantity of antibodies (serological titre), that bind the *T. gondii* P30 antigen coated in microwells, was performed measuring the optical densities (OD) of the colorimetric reaction (spectrophotometer—450 nm).Additionally, results of each ELISA plate were further validated. In detail, if the mean value of the positive control ODs (OD_{pc}) was greater than 0.350 ($OD_{pc} > 0.350$) and the ratio of the mean OD values of the positive and negative controls (OD_{pc} and OD_{nc}) was greater than 3 ($OD_{pc}/OD_{nc} > 3$), the test was considered conclusive. The results were interpreted applying the supplied formula

$$\frac{S}{P}\% = \left(\frac{(\text{ODsample} - \text{ODnc})}{(\text{ODpc} - \text{ODnc})}\right) x\,100$$

and samples with S/P% $\geq$ 50% were classified as positive. As in this study we aimed to include serological titres in statistical analyses, in order to compare values from different ELISA plates, each serological titre was normalised for the correspondent $\text{cutoff}_{50\%\ s/p}$ value applying the following formula:

$$\text{Normalisedserologicaltitre} = \frac{(\text{serologicaltitre} - \text{cutoff}\,50\%\text{value})}{\text{cutoff}\,50\%\text{value}}$$

This transformation was graphically checked in order to verify the correspondence between the normalised value and the related S/P% of each sample.

Statistical analysis

The data were analysed through Generalized Linear Models (GLMs). This analytical approach was chosen since it is robust towards potential analytic criticalness of unbalanced sample size and further stratifications in categories (age classes, hinds, etc.). The analyses were undertaken using SPSS Statistic 20.0® software; values were significant when $p<0.05$.

Factors affecting *T. gondii* infection

We used a binomial GLM to define the effects of host sex, age, body condition (KFI) and area on the probability of being seropositive to *T. gondii*. To investigate the effects of the same explanatory variables on infection dynamics, we modelled serological titres of seropositive subjects using a GLM with Gaussian distribution.

T. gondii effects on female fertility

The effect of *T. gondii* on female fertility was assessed trough two GLMs. Firstly we used a binomial GLM in order to define the effects of serological titres, age, body condition (KFI) and area on the probability of being pregnant. Then, through a Gaussian GLM we investigated the effects of serological titres, age, body condition(KFI), area, lactation status and sampling day on the physiological foetal development (gestational ages).

All models were initially fitted with all their first order interactions and the minimal models were obtained simplifying by backward selection based on AIC and AICC values to eliminate factors and variables that did not contribute significantly to the explanatory power of the model. Post-hoc analysis (Sidak test) was performed when statistically significant factors or interactions were detected.

Results

Factors affecting *T*. *gondii* infection

Red deer showed an overall prevalence(p) of 39.5% (95% CI: 29–50).The seropositivity was significantly influenced by age class (S2 Table), with calves having a lower probability of being infected (p = 4.8%, 95% CI: 4.3–14) than yearling (p = 52.4%, 95%CI: 31–73.8) (Sidak test p = 0.01) and adult (p = 51.3%, 95%CI: 35.6–67) (Sidak test p = 0.013) red deer. No significant difference was recorded between the two latter age classes (Sidak test $p>0.05$). No significant effect of the area was recorded (S2 Table) while sex and KFI were not retained in the minimal model.

Serological titres of positive red deer were significantly higher in yearlings than in adults (S3 Table). Area, sex and KFI were not retained in the minimal model.

T. *gondii* effects on female fertility

The probability of being pregnant was not influenced by any of the factors included in this analysis ($p>0.05$).

The foetal development (gestational ages) were estimated to vary from 25 to 68 days. Statistical analysis showed a significant positive effect of sampling day (S4 Table) and of KFI (S4 Table and Fig 2) on foetal development. Moreover, serological titres influenced the foetal development and this effect differed between area and female age class (S4 Table). In particular, in area 1 “low anthropised” a negative effect of increasing serological titres on foetal development was recorded in females of each age class (S4 Table and Fig 3) while in area 2 “high anthropised” the negative effect of serological titres was registered on foetal development in 2–3 year-old females (S4 Table and Fig 3) but not in other age classes (S4 Table and Fig 3).

Discussion

The present study showed high seroprevalence of *T*. *gondii* infection in red deer with seropositive individuals concentrated in older age classes supporting horizontal transmission as the main route of infection. Moreover *T*. *gondii* serological titres showed no association with pregnancy status, but a relation between females’ serological titres and a lower foetal development emerged, suggesting potential effects of the parasite infection on deer reproduction.

In the study population calves did not contract *T*. *gondii* infection, except for one female, while a widespread seropositivity to the parasite was recorded in red deer above one year of age. Our findings confirm the increase of seroprevalence with age highlighted in previous surveys and ascribed to a progressive postnatal acquisition of *T*. *gondii* related to the oral route of infection with older animals being exposed for a longer time [30, 47–51]. On the contrary in [13] no statistically significant differences in *T*. *gondii* prevalence was observed between red deer calves and older age classes. In the study area, vertical transmission therefore appears unlikely while the evidence for horizontal transmission suggests that cats are the most likely principal source of environmental contamination (shedding infecting oocysts). In Central Italian Alps other felids, as lynx (*Lynx lynx*), are indeed only sporadic. However, the equal *T*. *gondii* prevalence observed between the two sampling areas, despite the different anthropisation

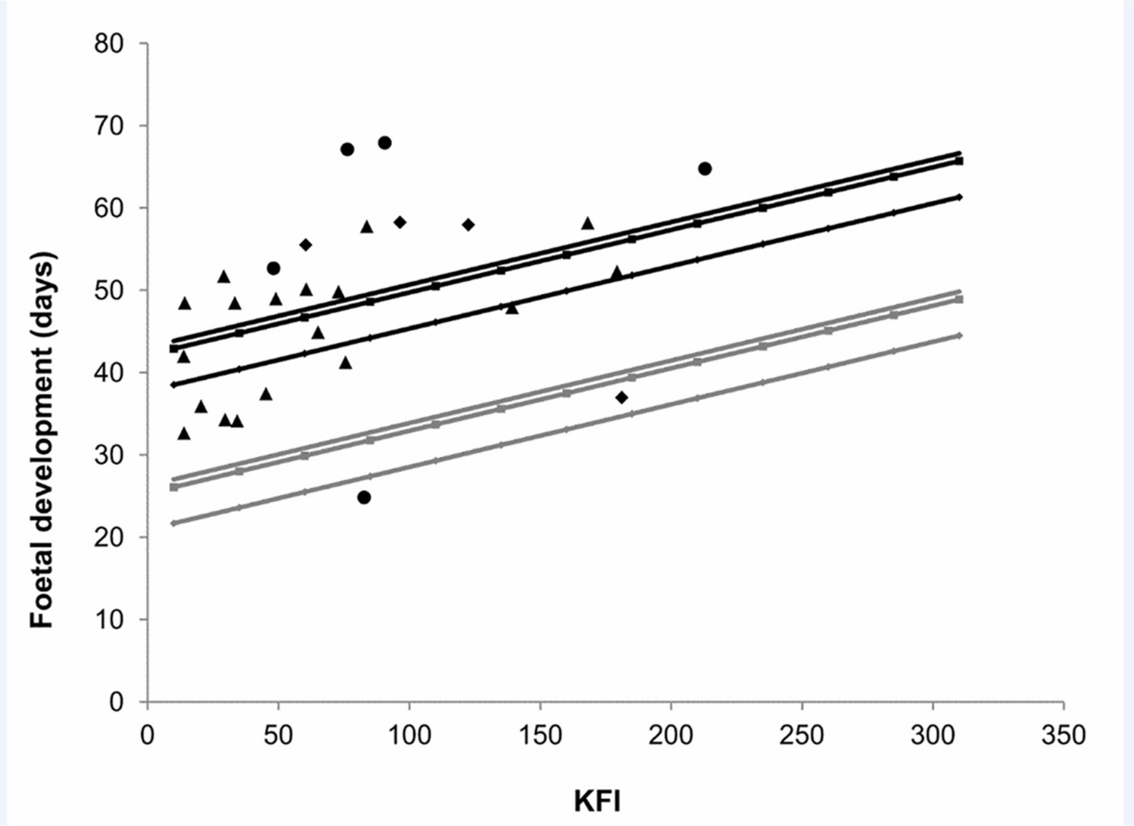

Fig 2. Effect of Kidney Fat Index (KFI) on foetal development (in days). Dots represent observed values (rhombus: 2–3 year-old females, circles: 4–6 year-old females; triangles: >7 year-old females) while lines represent the values predicted by the selected model (light grey line: area 1; black line: area 2).

doi:10.1371/journal.pone.0138472.g002

levels, suggests that the environmental contamination is poorly sensitive to the domestic cat density and is determined by other factors such as long lasting oocysts infectivity [52].Moreover the stable prevalence between yearling and adult individuals, with higher serological titres observed in the former, leads to hypothesise two alternative infection processes. The first one suggests new infections with the acquisition of long-lasting immunity in yearling individuals and old infections with low serological titres in adult red deer. As adults seem not to contract new infections, an age-related change in susceptibility/exposure appears. The alternative process leads to hypothesise that adult age class can quickly get rid of new infections (i.e. recovery/seroconvertion) providing stable prevalence and low serological titres. An age-related changes in host response to the infection (i.e. developing pathological symptoms, recovery/serocorvertion) must be therefore considered. While with our data we cannot discriminate which mechanism is really occurring in our population, [30] supposed that red deer acquired the infection only temporarily getting rid of it in early adulthood supporting our second hypothesis. However, the suggested age-related change in rate of infection seems to be confirmed by *T. gondii* effects on foetal development which changed with female age. The protozoan appears not to directly affect the probability of being pregnant, unlike what was supposed in goats [53], but could indirectly affect red deer fertility impairing foetal development. In particular, this finding could be the consequence of an infection acquired before the breeding season which influenced females' fertility by delaying mating or pregnancy or decreasing the foetal development. Young females could be more affected by *T. gondii* since they were still building up immunity against the parasite, without a complete resistance, and this could impair their foetal development.

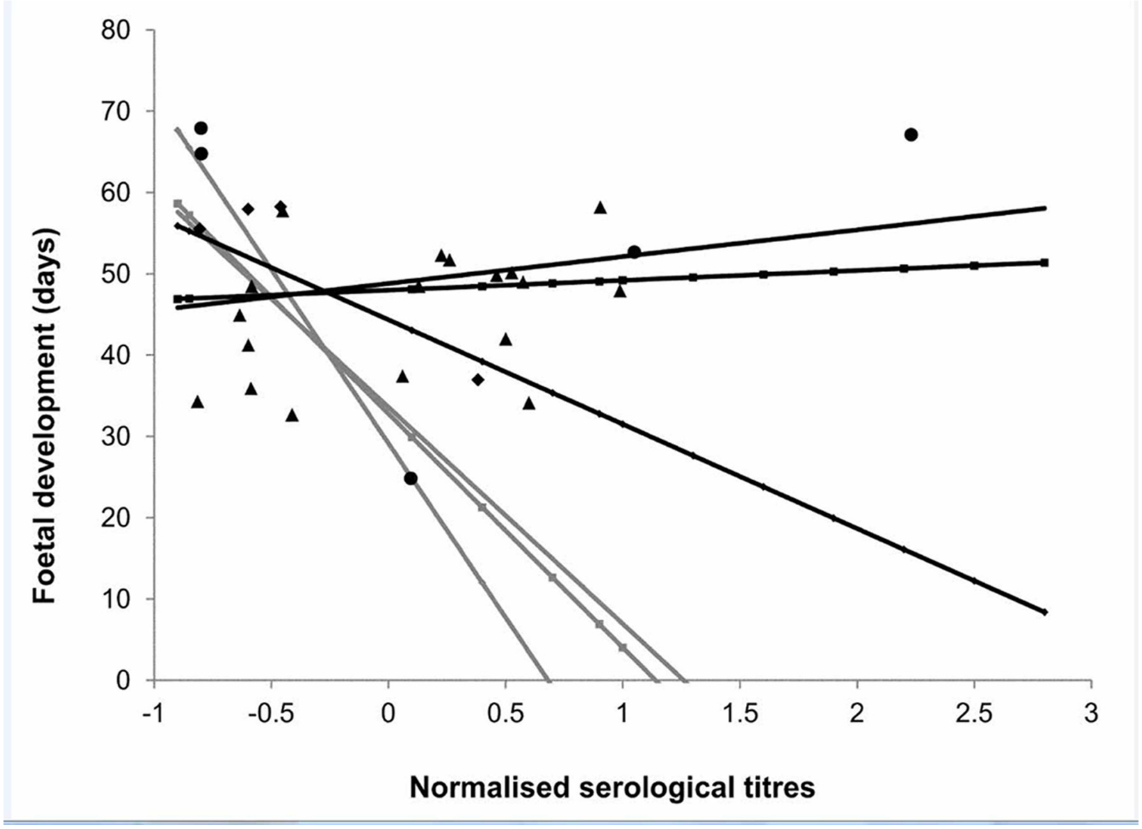

Fig 3. Effect of serological titres on foetal development (in days). Dots represent observed values (rhombus: 2–3 year-old females, circles: 4–6 year-old females; triangles: >7 year-old females); lines represent the values predicted by the selected model (light grey line: area 1; black line: area 2).

doi:10.1371/journal.pone.0138472.g003

However, in area 1 the *T. gondii* effect emerged even in middle-aged (4–6 year-old) and older (>7 year-old) females pointing out their higher susceptibility compared to same-aged females of area 2. Therefore, in older age classes *T. gondii* effects on foetal development appears to change with females' area. In particular, a role of habitat characteristics of area 1 in affecting hinds' physical condition and hence in impairing even older females' response to the protozoan could be emphasized. Although the current data don't allow us to assess the real mechanism which is occurring, Authors [54] have shown that hinds' home range area (quality and quantity of forage, density, etc.) can have important effects on most aspects of their reproductive performances supporting our hypothesis. Therefore, future analyses investigating habitat characteristics of the two areas should be carried out in order to evaluate their actual effects on hinds and to establish a relation between these data and the emerged impact on females' response to *T. gondii*.

Here new aspects of infection dynamics and effects of *T. gondii* were recorded in free-ranging red deer. Although our sample size and its further stratification into categories can appear contained, the sampling power was adequate to highlight the effects of the investigated factors excluding the occurrence of Type II error (i.e. not detection of effects when present). Additionally, sampling these free-ranging red deer from a National Park represents a great opportunity to achieve information about the host-parasite relationships in an unmanaged population. Due to the sampling coming from a single population, the results obtained in the present analyses should be however taken with care before being extended to larger environmental contexts. As this regards, experimental infections in farmed or captive-reared red deer can be further

powerful tools for providing additional information on the effects of parasites on wildlife populations, as highlighted by [55].

Conclusions

Our results showed high seroprevalence of *T. gondii* infection in older age classes and no infection, except for one female, in calves. Horizontal transmission appears, therefore, to be the main route of infection with feral and semi-domestic cats being the likely principal source of environmental contamination. Two alternative age-related *T. gondii*infection processes emerged and should be further investigated to discriminate which mechanism is really occurring. The pathogen seems not to prevent females becoming pregnant suggesting the resistance of this species to *T. gondii*-associated abortion. Nonetheless the negative effects on foetal development demonstrate that the parasite could have an indirect impact on red deer reproduction and thus, potentially, on population dynamics. In particular, young females of both areas appear susceptible to *T. gondii* effects confirming the suggested age-related change in rate of infection. On the other hand the impact on reproduction emerged only in middle-aged and older females from area 1 suggests an area-related change in *T. gondii* effects on older age classes supporting that environment can modulate the host-parasite interaction.

Supporting Information

S1 Table. Overall red deer sera sample size according to sex and age class.
(DOCX)

S2 Table. Minimal model of factors influencing the probability for red deer to be infected.
(DOCX)

S3 Table. Minimal model describing factors influencing serological titres of seropositive red deer.
(DOCX)

S4 Table. Minimal model of factors affecting foetal development.
(DOCX)

S1 Text. Granted permission for the publication of Fig 1.
(PDF)

Acknowledgments

We thank Alessandro Gugiatti and Andrea Zanoli for their support in the field, IlariaMarangi and Joanne Lello for their valuable contributions that improved the manuscript.

Author Contributions

Conceived and designed the experiments: N. Formenti PL N. Ferrari. Performed the experiments: N. Formenti TT AG. Analyzed the data: N. Formenti LP N. Ferrari. Contributed reagents/materials/analysis tools: AG LP PL. Wrote the paper: N. Formenti TT LP AG PL N. Ferrari.

References

1. Hudson PJ, Dobson AP. Macroparasites: observed patterns in naturally fluctuating animal population. In: Grenfell BT, Dobson AP, editors. Ecology of infectious disease in natural population. Cambridge University Press; 1995. pp. 146–176.

2. Citterio CV, Caslini C, Milani F, Sala M, Ferrari N, Lanfranchi P. Abomasal nematode community in an alpine chamois (*Rupicapra r*. *rupicapra*) population before and after a die-off. J Parasitol. 2006; 92: 918–927. PMID: 17152929
3. Turchetto S, Obber F, Permunian R, Vendrami S, Lorenzetto M, Ferré N, et al. Spatial and temporal explorative analysis of sarcoptic mange in Alpine chamois (*Rupicapra r*. *rupicapra*). Hystrix.2014; 25: 25–30.
4. Albon SD, Stien A, Irvine RJ, Langvatn R, Ropstad E, Halvorsen O. The role of parasites in the dynamics of a reindeer population. Proc R SocLond B. 2002; 269: 1625–1632.
5. Lello J, Boag B, Hudson PJ. The effect of single and concomitant pathogen infections on condition and fecundity of the wild rabbit (*Oryctolaguscuniculus*). Int J Parasitol. 2005; 35: 1509–1515. PMID: 16051247
6. Hudson PJ, Newborn D, Dobson AP. Regulation and stability of a free-living host–parasite system: *Trichostrongylus tenuis* in red grouse. I. Monitoring and parasite reduction experiments. J Anim Ecol.1992; 61: 477–486.
7. Anderson ML, Barr BC, Conrad PA. Protozoal causes of reproductive failure in domestic ruminants. Vet Clin N Am-Food A. 1994; 10: 439–461.
8. Fox MT. Pathophysiology of infection with gastrointestinal nematodes in domestic ruminants: recent developments. Vet Parasitol.1997; 72: 285–308. PMID: 9460203
9. Alberti EG, Zanzani SA, Gazzonis AL, Zanatta G, Bruni G, Villa M, et al.Effects of gastrointestinal infections caused by nematodes on milk production in goats in a mountain ecosystem: Comparison between a cosmopolite and a local breed. Small Ruminant Res. 2014; 120: 155–163.
10. Dubey JP. Isolation of *Toxoplasma gondii*from a Naturally Infected Beef Cow. J Parasitol. 1992; 78: 151–153. PMID: 1738059
11. Cabezón O, García-Bocanegra I, Molina-López R, Marco I, Blanco JM, Höfle U, et al. Seropositivity and risk factors associated with *Toxoplasma gondii* infection in wild birds from Spain. PLoS ONE. 2011; 6: 1–7.
12. Gibson AK, Raverty S, Lambourn DM, Huggins J, Magargal SL, Grigg ME. Polyparasitismis associated with increased disease severity in *Toxoplasma gondii*infected marine sentinel species. PLoS ONE. 2011; 5: 1–13.
13. Gauss CBL, Dubey JP, Vidal D, Cabezón O, Ruiz-Fons F, Vicente J, et al. Prevalence of *Toxoplasma gondii* antibodies in red deer (*Cervuselaphus*) and other wild ruminants from Spain. Vet Parasitol. 2006; 136: 193–200. PMID: 16359801
14. Sobrino R, Cabezón O, Millán J, Pabón M, Arnal MC, Luco DF, et al. Seroprevalence of *Toxoplasma gondii* antibodies in wild carnivores from Spain. Vet Parasitol. 2007; 148: 187–192. PMID: 17689869
15. Gamarra JA, Cabezón O, Pabón M, Arnal MC, Luco DF, Dubey JP, et al. Prevalence of antibodies against *Toxoplasma gondii*in roe deer from Spain. Vet Parasitol. 2008; 153: 152–156. doi: 10.1016/j.vetpar.2008.01.028 PMID: 18316161
16. Jokelainen P, Näreaho A, Knaapi S, Oksanen A, Rikula C, Sukura A. *Toxoplasma gondii* in wild cervids and sheep in Finland: North-south gradient in seroprevalence. Vet Parasitol. 2010; 171: 331–336. doi: 10.1016/j.vetpar.2010.04.008 PMID: 20434266
17. Jokelainen P, Isomursu M, Näreaho A, Oksanen A. Natural *Toxoplasma gondii* infections in European brown hares and mountain hares in Finland: proportional mortality rate, antibody prevalence, and genetic characterization. J Wildl Dis.2011; 47: 154–163. PMID: 21270004
18. Opsteegh M, Swart A, Fonville M, Dekkers L, van der Giessen J. Age-Related *Toxoplasma gondii* Seroprevalence in Dutch Wild Boar Inconsistent with Lifelong Persistence of Antibodies. PLoS ONE. 2011; 6: 1–6.
19. Duncanson P, Terry RS, Smith JE, Hide G. High levels of congenital transmission of *Toxoplasma gondii* in a commercial sheep flock. Int J Parasitol. 2001; 31: 1699–1703. PMID: 11730799
20. Masala G, Porcu R, Madau L, Tanda A, Ibba B, Satta G, et al. Survey of ovine and caprine toxoplasmosis by IFAT and PCR assays in Sardinia, Italy. Vet Parasitol. 2003; 117: 15–21. PMID: 14597274
21. Pereira-Bueno J, Quintanilla-Gozalo A, Pérez-Pérez V, Álvarez-García G, Collantes-Fernández E, Ortega-Mora LM. Evaluation of ovine abortion associated with *Toxoplasma gondii*in Spain by different diagnostic techniques. Vet Parasitol. 2004; 121: 33–43. PMID: 15110401
22. Morley EK, Williams RH, Hughes JM, Thomasson D, Terry RS, Duncanson P, et al. Evidence that primary infection of Charollais sheep with *Toxoplasma gondii* may not prevent foetal infection and abortion in subsequent lambings. Parasitology. 2008; 135: 169–173. PMID: 17922930
23. Cook AJC, Gilbert RE, Buffolano W, Zufferey J, Petersen E, Jenum PA, et al. Sources of Toxoplasma infection in pregnant women: European multicentre casecontrol study. BMJ. 2000; 321: 142–147. PMID: 10894691

24. European Food Safety Authority. Surveillance and monitoring of *Toxoplasma* in humans, food and animals. Scientific Opinion of the Panel on Biological Hazards. The EFSA Journal. 2007; 583: 28–64.

25. Robert-Gangneux F, Dardé ML. Epidemiology of and Diagnostic Strategies for Toxoplasmosis. ClinMicrobiol. 2012; 25: 264–296.

26. Huong LTT, Ljungstrom BL, Uggla A, Bjorkman C. Prevalence of antibodies to *Neosporacaninum*and *Toxoplasma gondii*in cattle and water buffaloes in southern Vietnam. Vet Parasitol. 1998; 75: 53–57. PMID: 9566094

27. Rossi GF, Cabral DD, Ribeiro DP, Pajuaba ACAM, Corrêa RR, Moreira RQ, et al. Evaluation of *Toxoplasma gondii* and *Neosporacaninum* infections in sheep from Uberlândia, Minas Gerais State, Brazil, by different serological methods. Vet Parasitol. 2011; 175: 252–259. doi: 10.1016/j.vetpar.2010.10.017 PMID: 21075529

28. Kapperud G, Jenum PA, Stray-Pedersen B, Melby KK, Eskild A, Eng J. Risk factors for *Toxoplasma gondii*infection in pregnancy results of a prospective case-control study in Norway. Am J Epidemiol. 1996; 144: 405–412. PMID: 8712198

29. Hejlíček K, Literák I, Nezval J. Toxoplasmosis in wild mammals from the Czech Republic. J Wildl Dis. 1997; 33: 480–485. PMID: 9249693

30. Vikøren T,Tharaldsen J, Fredriksen B, Handeland K. Prevalence of *Toxoplasma gondii*antibodies in wild red deer, roe deer, moose, and reindeer from Norway. Vet Parasitol.2004; 120: 159–169. PMID: 15041091

31. Gaffuri A, Giacometti M, Tranquillo VM, Magnino S, Cordioli P, Lanfranchi P. Serosurvey of roe deer, chamois and domestic sheep in the central Italian Alps. J Wildl Dis. 2006; 42: 685–690. PMID: 17092903

32. Aubert D, Ajzenberg D, Richomme C, Gilot-Fromont E, Terrier ME, de Gevigney C, et al. Molecular and biological characteristics of *Toxoplasma gondii* isolates from wildlife in France. Vet Parasitol.2010; 171: 346–349. doi: 10.1016/j.vetpar.2010.03.033 PMID: 20417034

33. Magnino S, Frasnelli M, Fabbi M, Bianchi A, Zanoni MG, Merialdi G, et al. The monitoring of selected zoonotic diseases of wildlife in Lombardy and Emilia-Romagna, northern Italy. In: Paulsen P, Bauer A, Vodnansky M, Winkelmayer R, Smulders FJM, editors.Game meat hygiene in focus. Microbiology, epidemiology, risk analysis and quality assurance. The Netherlands: Wageningen Academic Publishers;2011. pp. 223–244.

34. Beral M, Rossi S, Aubert D, Gasqui P, Terrier M-E, Klein F, et al. Environmental factors associated with the seroprevalence of *Toxoplasma gondii* in Wild Boars (*Sus scrofa*), France. EcoHealth. 2012; 9: 303–309. doi: 10.1007/s10393-012-0786-2 PMID: 22820902

35. Dubey JP, Velmurugan GV, Ulrich V, Gill J, Carstensen M, Sundar N, et al. Transplacental toxoplasmosis in naturally-infected white-tailed deer: Isolation and genetic characterisation of *Toxoplasma gondii* from foetuses of different gestational ages. Int J Parasitol. 2008; 38: 1057–1063. doi: 10.1016/j.ijpara.2007.11.010 PMID: 18187136

36. Elmore SA, Jenkins EJ, Huyvaert KP, Polley L, Root JJ, Moore CG. *Toxoplasma gondii* in Circumpolar People and Wildlife. Vector Borne Zoonotic Dis. 2012; 12: 1–9. doi: 10.1089/vbz.2011.0705 PMID: 21995261

37. Albon SD, Mitchell B, Huby BJ, Brown D. Fertility in female Red deer (*Cervuselaphus*): the effects of body composition, age and reproductive status. J Zool.1986; 209: 447–460.

38. Kojola I, Eloranta E. Influences of Maternal Body Weight, Age, and Parity on Sex Ratio in Semidomesticated Reindeer (*Rangifer t. tarandus*). Evolution. 1989; 43: 1331–1336.

39. Adam CL, McDonald I, Moir CE, Pennie K. Foetal development in red deer (*Cervuselaphus*) 1. Growth of the foetus and associated tissues. Anim Prod. 1988; 46: 131–138.

40. Chong C-K, Jeong W, Kim H-Y, An D-J, Jeoung H-Y, Ryu J-E, et al. Development and Clinical Evaluation of a Rapid Serodiagnostic Test for Toxoplasmosis of Cats Using Recombinant SAG1 Antigen. Korean J Parasitol.2011; 49: 207–212. doi: 10.3347/kjp.2011.49.3.207 PMID: 22072819

41. Shadfar S, Shabestari A, Zendeh MB, Gasemi B, Zamzam SH. Evaluation of *Toxoplasma gondii* IgG Antibodies in Stray and Household Dogs by Elisa. Global Veterinaria.2012; 9: 117–122.

42. Gebremedhin EZ, Agonafir A, Tessema TS, Tilahun G, Medhin G, Vitale M, et al. Some risk factors for reproductive failures and contribution of *Toxoplasma gondii* infection in sheep and goats of Central Ethiopia: A cross-sectional study. Res Vet Sci. 2013; 95: 894–900. doi: 10.1016/j.rvsc.2013.08.007 PMID: 24011595

43. Dărăbus G, Afrenie M, Hotea I, Imre M, Morariu S. Endoparasites in mammals from seven zoological gardens in Romania. J Zoo Wildl Med. 2014; 45: 239–246. PMID: 25000683

44. Gebremedhin EZ, Yunus HA, Tesfamaryam G, Tessema TS, Dawo F, Terefe G, et al. First report of *Toxoplasma gondii* in camels (*Camelusdromedarius*) in Ethiopia: bioassay and seroepidemiological investigation. BMC Vet Res. 2014; 10:222. doi: 10.1186/s12917-014-0222-7 PMID: 25266944

45. Hernandez M, Gomez-Laguna J, Tarradas C, Luque I, Garcıa-Valverde R, Reguillo L, et al. A serological Survey of *Brucella* spp., *Salmonella* spp., *Toxoplasma gondii* and *Trichinella* spp. in Iberian Fattening Pigs Reared in Free-Range Systems. TransboundEmerg Dis. 2014; 61: 477–481.

46. Onyiche TE, Ademola IO. Seroprevalence of anti-*Toxoplasma gondii* antibodies in cattle and pigs in Ibadan, Nigeria. J ParasitDis. 2015; 39: 309–314.

47. van der Puije WNA, Bosompem KM, Canacoo EA, Wastling JM, Akanmori BD. The prevalence of anti-*Toxoplasma gondii*antibodies in Ghanaian sheep and goats. Acta Trop. 2000; 76: 21–26. PMID: 10913761

48. Figliuolo LPC, Rodrigues AAR, Viana RB, Aguiar DM, Kasai N, Gennari SM. Prevalence of anti-*Toxoplasma gondii*and anti-*Neosporacaninum*antibodies in goat from São Paulo State, Brazil. Small Ruminant Res. 2004; 55: 29–32.

49. Jittapalapong S, Sangvaranond A, Pinyopanuwat N, Chimnoi W, Khachaeram W, Koizumi S, et al. Seroprevalence of *Toxoplasma gondii* infection in domestic goats in Satun Province, Thailand. Vet Parasitol. 2005; 127: 17–22. PMID: 15619370

50. Clementino MM, Souza MF, Andrade Neto VF. Seroprevalence and *Toxoplasma gondii*-IgG avidity in sheep from Lajes, Brazil. Vet Parasitol. 2007; 146: 199–203. PMID: 17418950

51. Chikweto A, Kumthekar S, Tiwari K, Nyack B, Deokar MS, Stratton G, et al.Seroprevalence of *Toxoplasma gondii* in Pigs, Sheep, Goats, and Cattle From Grenada and Carriacou, West Indies. J Parasitol. 2011; 97: 950–951. doi: 10.1645/GE-2811.1 PMID: 21506801

52. Kijlstra A, Jongert E. Control of the risk of human toxoplasmosis transmitted by meat. Int J Parasitol. 2008; 38: 1359–1370. doi: 10.1016/j.ijpara.2008.06.002 PMID: 18694755

53. Mišurová L, Svobodová V, Pavlata L, Dvořák R. Titres of Specific Antibodies against *Toxoplasma gondii*in Goats and their Kids. Acta Vet Brno. 2009; 78: 259–266.

54. Guinness FE, Albon SD, Clutton-Brock TH. Factors affecting reproduction in red deer (*Cervuselaphus*) hinds on Rhum. J ReprodFert. 1978; 54: 325–334.

55. Pedersen AB, Fenton A. The role of antiparasite treatment experiments in assessing the impact of parasites on wildlife. Trends Parasitol. 2015; 31: 200–211. doi: 10.1016/j.pt.2015.02.004 PMID: 25778845
